# Tumor stromal nicotinamide N-methyltransferase overexpression as a prognostic biomarker for poor clinical outcome in early-stage colorectal cancer

**DOI:** 10.1038/s41598-022-06772-w

**Published:** 2022-02-17

**Authors:** Makiko Ogawa, Atsushi Tanaka, Kei Namba, Jinru Shia, Julia Y. Wang, Michael H. A. Roehrl

**Affiliations:** 1 Department of Pathology and Laboratory Medicine, Memorial Sloan Kettering Cancer Center, NY New York, USA; 2 Human Oncology and Pathogenesis Program, Memorial Sloan Kettering Cancer Center, New York, NY USA; 3 Department of Pathology, Graduate School of Medicine, University of Tokyo, Tokyo, Japan; 4 Department of Thoracic Surgery and Breast and Endocrine Surgery, Okayama University Graduate School of Medicine, Dentistry, and Pharmaceutical Sciences, Okayama, Japan; 5 Curandis, New York, NY USA; 6 Weill Cornell Medicine Graduate School of Medical Sciences, New York, NY USA

**Keywords:** Gastrointestinal cancer, Tumour biomarkers, Predictive markers, Translational research

## Abstract

In a quest for prognostic biomarkers in early-stage colorectal cancer, we investigated NNMT (nicotinamide N-methyltransferase) in large cohorts of patients. Immunohistochemical examination of 679 patients illustrates that NNMT protein is predominantly expressed in the cancer stroma at varying levels, and about 20% of cancer tissues overexpress NNMT when compared to levels observed in normal colorectal mucosa. Clinical correlation analyses of 572 patients with early-stage cancers reveal that NNMT protein overexpression is significantly associated with shorter overall and disease-free survival, but no such correlation is found in late-stage colorectal cancer. Analyses of TCGA and CPTAC colorectal cancer cohorts show that NNMT mRNA expression is positively correlated with protein levels, is significantly higher in CIMP-high or MSI subtypes than in CIMP-low or MSS subtypes, and is positively correlated with its paralog INMT but not with its interaction partners such as PNMT, ADK, APP, ATF6, BMF, BRD4, CDC37, or CRYZ. In early-stage cancers, NNMT expression is higher in BRAF-mutated than in BRAF wild type tumors but is not affected by KRAS or PIK3CA mutation status. As a cancer stromal protein with important roles in metabolism and cancer epigenetics, NNMT is emerging as a promising biomarker for risk stratification of early-stage cancers.

## Introduction

Colorectal cancer is among the most common cancers in both men and women^1^. In the United States, the lifetime risk of developing colorectal cancer is about 1 in 23 for men and 1 in 25 for women according to the American Cancer Society. The death rate from colorectal cancer has been dropping in recent years, partially due to increased screening efforts and early detection^2^. However, early onset disease has been on the rise in younger patients who, typically, are not routinely screened by colonoscopy, and these patients are often diagnosed at an advanced stage which poses numerous unique challenges for cancer management^3^. Therefore, better cancer prevention and care calls for broadening cancer screening in the general population, which entails discovery and use of molecular biomarkers that are readily detectable in early-stage cancers.

One of potentially readily detectable markers is NNMT (nicotinamide N-methyltransferase)^4^. NNMT is overexpressed in a variety of cancers^5–19^. Elevated levels of NNMT have been found in sera from patients with colorectal cancer, although NNMT is known as a cytoplasmic protein and not predicted to be secreted^20^. NNMT is a metabolic enzyme that methylates nicotinamide (niacinamide) using the universal methyl donor S-adenosyl methionine (SAM)^21–23^. When overexpressed, NNMT impairs the methylation balance of cancer cells by consuming methyl units, changes protein and gene methylation landscapes, and may result in hypomethylated histone and alteration of the epigenetic state of cancer cells^6^.

Nicotinamide is a member of the vitamin B3 family compounds which are precursors of nicotinamide adenine dinucleotide (NAD+) and its phosphorylated parent NADP+. The redox pairs of NAD(P)+ and NAD(P)H are central to metabolism, serving as cofactors in many redox enzymes. Nicotinamide methylation by NNMT is the major pathway for its degradation and secretion in the urine. NNMT also catalyzes N-methylation of nicotinamide-similar pyridines, which is important for transformation of many drugs and xenobiotic compounds^24^. NNMT has been identified as a master metabolic regulator of cancer progression in high-grade serous ovarian carcinoma^25^. NNMT overexpression has been reported to decrease drug sensitivity and enhance chemoresistance in breast cancer, esophageal squamous cell carcinoma, and cell lines of colorectal cancer and melanoma^26–29^. With crucial roles in metabolism and methylation, NNMT is emerging as a key intersection point between cellular metabolism and epigenetic gene regulation in cancer^6^. In this study, we sought to investigate whether NNMT is a prognostic marker in early-stage colorectal cancer.

## Materials and methods

### Clinical case selection and pathological data

Colorectal cancer tissue specimens from 679 patients were obtained from the pathology archives of Memorial Sloan Kettering Cancer Center (MSKCC). The cohort comprises 572 cases of early stage (AJCC stages I or II) and 107 cases of late stage (AJCC stages III or IV). The study has been approved by MSKCC’s Institutional Review Board (IRB), and clinical data were acquired retrospectively in an anonymized manner. All experiments were performed in accordance with relevant guidelines and regulations. Due to the de-identified and retrospective nature of the study, the IRB has determined that informed consent was waived. Clinical parameters, including patient age, treatment history, recurrence, and survival status, were retrieved from medical records. Histologic features and other clinicopathological parameters of all samples were re-verified independently by two gastrointestinal subspecialty pathologists on our team (AT and MHAR).

### Tissue microarray construction

Tissue microarrays were constructed from the 679 colorectal tumors. All archival tissue specimens had been fixed with formalin and embedded in paraffin blocks. Three 2-mm tissue cores were drilled out from each donor paraffin tissue block and transferred to tissue array blocks using a TMA Grand Master robot (3DHistech). The cored areas were defined by a certified pathologist for each case and tissue block and included tumor tissue as well as paired normal mucosal tissue.

### Immunohistochemistry (IHC)

The tissue microarray blocks were cut into 4-µm sections. Paraffin was removed with xylene, and antigens were retrieved by BOND epitope retrieval solution 2 (EDTA buffer, pH 9.0) performed on the Leica BOND RX slide stainer for 30 min at 100 °C. Tissue sections were incubated with NNMT-specific polyclonal antibodies (HPA059180, 1:200, Atlas Antibodies, Sigma) for 30 min. They were followed by visualization with the Leica Bond detection kit (DS9800).

### Immunohistochemical scoring

Stained IHC tissue slides were evaluated independently by two pathologists without knowledge of the patients’ clinical information. Each tissue section was scored by counting the number of lamina propria stromal cells staining positively for NNMT protein (staining intensity ≥1+) relative to the total number of evaluated stromal cells. A minimum of 1000 stromal cells was evaluated per tissue sample. A tissue sample was considered positive for NNMT expression (“high”) when >50% of stromal cells showed positive cytoplasmic staining, otherwise negative (“low”). This two-tiered scoring approach was used to maximize the number of patients in each category and to achieve optimal statistical power to detect any group differences.

### cBioPortal dataset analysis

Sequencing results and relevant clinical information were downloaded from cBioPortal^30,31^. Pan-cancer cell line data from the cell line encyclopedia was used^32^.Two colorectal cancer cohorts (comprising 594 cases in one and 274 cases in another cohort) from the Cancer Genome Atlas (TCGA)^33,34^ and a colorectal cancer cohort from the Clinical Proteomics Tumor Assessment Consortium (CPTAC)^35^ were analyzed for gene and protein expression levels of NNMT, KRAS, BRAF, PIK3AC, INMT, PNMT, ADK, APP, ATF6, BMF, BRD4, CDC37, and CRYZ. NNMT expression in colorectal cancer subtypes was compared, including CIMP-high (CpG island methylator phenotype) vs. CIMP-low and MSI vs. MSS.

### Statistical analysis

Categorical variables were compared using Fisher’s exact test. Survival analyses were conducted using the Kaplan–Meier method and compared by a log-rank test. Multivariate analyses of prognostic factors were performed with logistic regression models by using factors that showed significant differences (*p* < 0.05) in univariate analyses. A backward elimination method was used to select variables for the final model. Correlation coefficients were calculated by the Spearman method. Statistical analyses were performed using JMP Pro 14 software (SAS).

## Results

### NNMT protein expression in colorectal cancer

**Figure 1:**
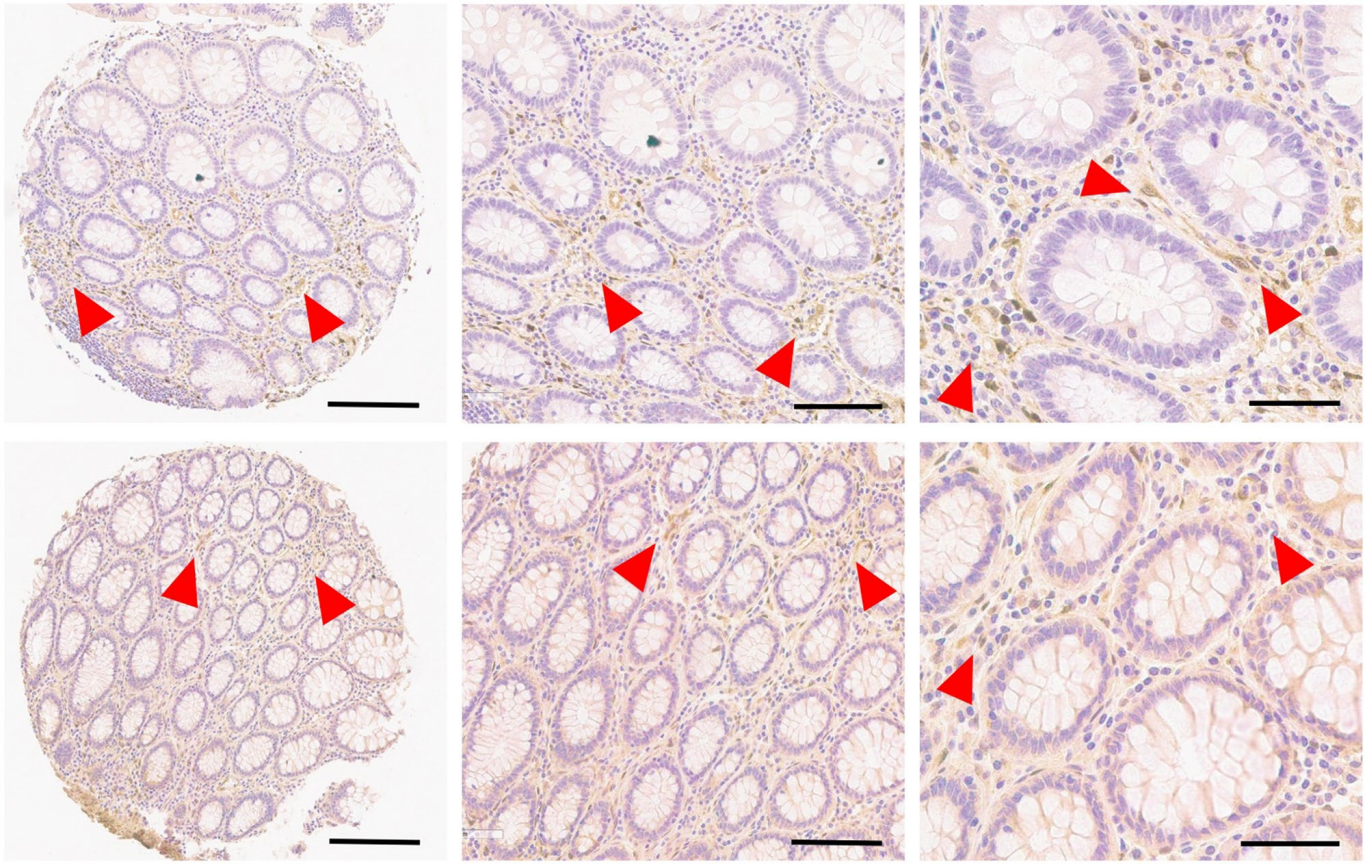
NNMT protein expression in normal (benign) colorectal mucosa detected by immunohistochemistry. NNMT expression is primarily stromal (arrowheads show examples). NNMT proteins are stained in brown, cell nuclei are counterstained in blue. Black bars and original magnifications: 400 µm/100 × (left panels), 200 µm/200 × (middle panels), and 100 µm/400 × (right panels).

In order to understand changes of NNMT protein expression in cancers, we first examined normal colonic and rectal mucosa by immunohistochemistry. In normal colorectal mucosa, NNMT protein expression is low to focally moderate and localized to the cytoplasm of stromal cells, while enterocytes in colonic crypt and surface epithelium show essentially negative to very low expression (Fig. 1).

**Figure 2:**
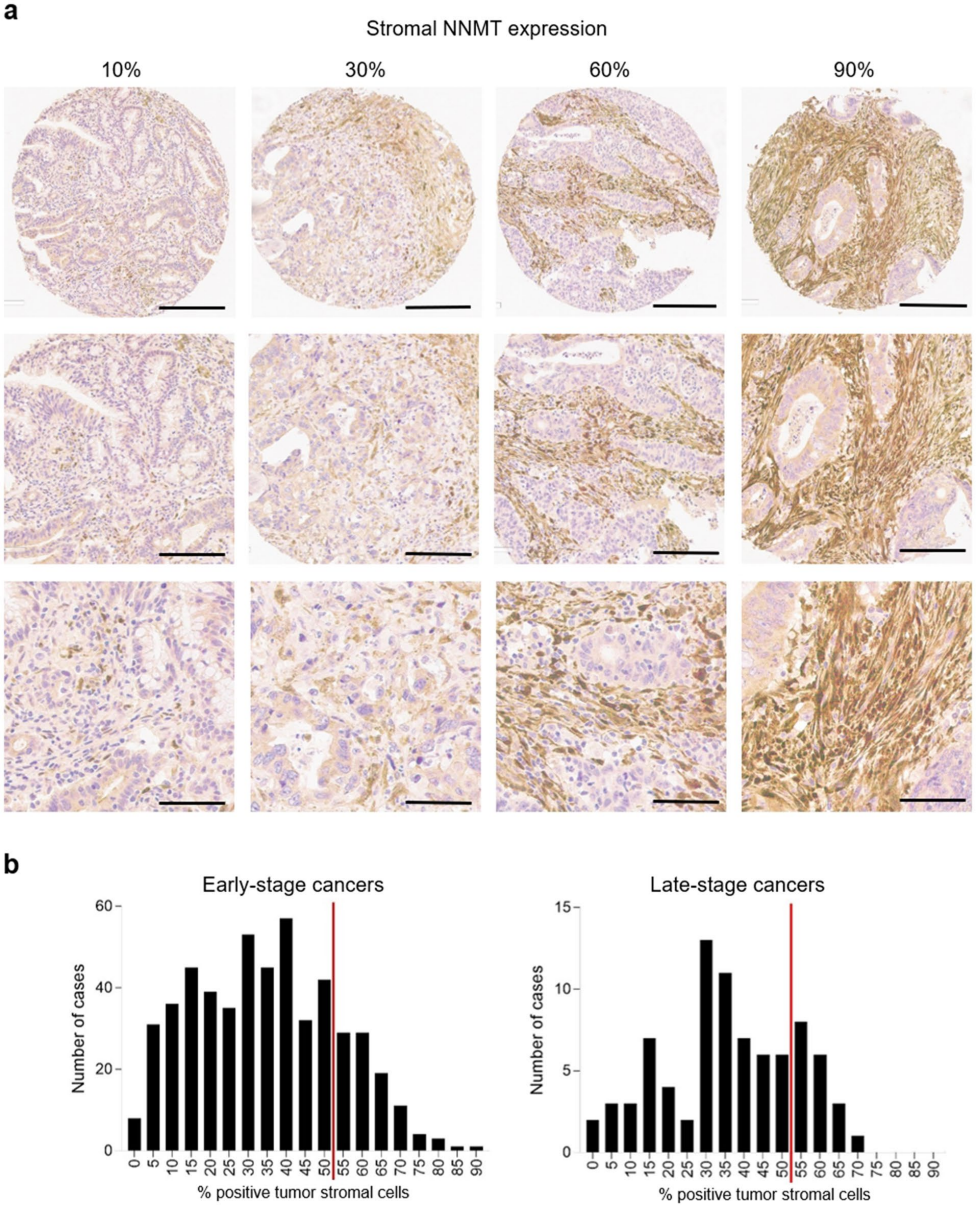
(**a**) Representative range of tumor stromal NNMT protein expression in four different patients with colorectal adenocarcinomas (10%, 30%, 60%, or 90% of tumor stromal expression, respectively). NNMT proteins are stained in brown. Cell nuclei are counterstained in blue. Top numbers indicate the percentage of tumor stromal cells staining positively. Black bars and original magnifications: 400 µm/100 × (top row), 200 µm/200 × (middle row), and 100 µm/400 × (bottom row). (**b**) NNMT expression distribution for 520 early-stage and 82 late-stage cancers. The high vs. low protein expression cutoff is shown by a vertical red line. Overexpression (right of red line) is defined as >50% of tumor stromal cells staining positively for NNMT.

In contrast, colorectal cancers show NNMT protein expression in tumor stroma that varies between individual patients (Fig. 2a), ranging from few positive tumor stromal cells to strong expression in most stromal cells. Similar to their benign enterocyte counterparts, invasive cancer cells, however, express no to very low levels of NNMT. We also examined *NNMT* mRNA expression levels in a variety of cancer cell lines from the cancer cell line encyclopedia^32,36,37^. *NNMT* mRNA expression is indeed very low in colorectal cancer cells, whereas, for example, kidney cancer or mesothelioma cells express much higher levels of NNMT (Supplemental Fig. 1a). Transcript and protein expression of NNMT correlate positively (Supplemental Fig. 1b).

To better quantitate NNMT expression levels, we investigated the immunohistochemical staining of tumor stroma using two well-annotated cohorts comprising a total of 679 colorectal cancer patients across stages I–IV (Table 1). We measured NNMT expression as the percentage of positive tumor stromal cells for each patient (Fig. 2b). The distribution histograms for both early-stage and late-stage cancers showed a broad distribution across our patient cohorts. We defined tumors with over 50% positive tumor stromal immunohistochemical staining as NNMT overexpressing (or high). The expression range distributions of early-stage and late-stage cohorts were similar. In the 572-case early-stage cancer cohort, 79.9% of the cases had low expression and 20.1% had high expression. In the 107-case late-stage cancer cohort, 81.3% had low expression and 18.7% had high expression.

**Table 1:** Clinicopathological characteristics of the colorectal cancer patient cohorts (total n = 679).

	Early-stage cohort	Late-stage cohort	Combined
**Total**	572	107	679
**Gender**			
Male	300 (52.4%)	51 (47.7%)	351 (51.7%)
Female	272 (47.6%)	56 (52.3%)	328 (48.3%)
**Age (years)**			
≤ 70	332 (58.0%)	99 (92.5%)	431 (63.5%)
> 70	240 (42.0%)	8 (7.5%)	248 (36.5%)
**Histology**			
Mucinous	44 (7.7%)	11 (10.3%)	55 (8.1%)
Not mucinous	528 (92.3%)	96 (89.7%)	624 (91.9%)
**Tumor differentiation**			
G1/G2	523 (91.4%)	91 (85.0%)	614 (90.4%)
G3	49 (8.6%)	16 (15.0%)	65 (9.6%)
**Location**			
Left	279 (48.8%)	70 (65.4%)	349 (51.4%)
Right	293 (51.2%)	37 (34.6%)	330 (48.6%)
**Lymphovascular invasion**			
Absent	495 (86.5%)	31 (29.0%)	526 (77.5%)
Present	77 (13.5%)	76 (71.0%)	153 (22.5%)
**Perineural invasion**			
Absent	543 (94.9%)	67 (62.6%)	610 (89.8%)
Present	29 (5.1%)	40 (37.4%)	69 (10.2%)
**Clinical stage**			
I	211 (36.9%)		211 (31.1%)
II	361 (63.1%)		361 (53.2%)
III		77 (72.0%)	77 (11.3%)
IV		30 (28.0%)	30 (4.4%)
**MMR status**			
Intact (MSS)	439 (76.7%)	93 (86.9%)	532 (78.4%)
Lost (MSI)	133 (23.3%)	14 (13.1%)	147 (21.6%)

### NNMT expression versus clinicopathological features

To investigate whether tumor stromal NNMT expression is associated with clinicopathological features of the cancer patients, we analyzed various parameters, including gender, age, tumor histology, tumor differentiation, and others (Table 2).

**Table 2:** Association of NNMT protein expression with clinicopathological features of colorectal cancer patients.

	NNMT in early-stage cancers (n = 572)			NNMT in late-stage cancers (n = 107)		
	Low 457 (79.9%)	High 115 (20.1%)	*p*-value*	Low 87 (81.3%)	High 20 (18.7%)	*p*-value*
**Gender**			1.0000			0.8098
Male	240 (80.0%)	60 (20.0%)		42 (82.4%)	9 (17.6%)	
Female	217 (79.8%)	55 (20.2%)		45 (80.4%)	11 (19.6%)	
**Age (years)**			0.4599			**0.0055**
≤ 70	269 (81.0%)	63 (19.0%)		84 (84.8%)	15 (15.2%)	
> 70	188 (78.3%)	52 (21.7%)		3 (37.5%)	5 (62.5%)	
**Histology**			0.6952			0.4272
Mucinous	34 (77.3%)	10 (22.7%)		8 (72.7%)	3 (27.3%)	
Not mucinous	423 (80.1%)	105 (19.9%)		79 (82.3%)	17 (17.7%)	
**Tumor differentiation**			0.4556			1.0000
G1/G2	420 (80.3%)	103 (19.7%)		74 (81.3%)	17 (18.7%)	
G3	37 (75.5%)	12 (24.5%)		13 (81.3%)	3 (18.7%)	
**Location**			0.6776			0.3046
Left	225 (80.6%)	54 (19.4%)		59 (84.3%)	11 (15.7%)	
Right	232 (79.2%)	61 (20.8%)		28 (75.7%)	9 (24.3%)	
**Lymphovascular invasion**			1.0000			0.7884
Absent	395 (79.8%)	100 (20.2%)		26 (83.9%)	5 (16.1%)	
Present	62 (80.5%)	15 (19.5%)		61 (80.3%)	15 (19.7%)	
**Perineural invasion**			0.8155			0.6094
Absent	433 (79.7%)	110 (20.3%)		53 (79.1%)	14 (20.9%)	
Present	24 (82.8%)	5 (17.2%)		34 (85.0%)	6 (15.0%)	
**Clinical stage**			**0.0001**			
I	186 (88.2%)	25 (11.8%)				
II	271 (75.1%)	90 (24.9%)				
**Clinical stage**						**0.0116**
III				58 (75.3%)	19 (24.7%)	
IV				29 (96.7%)	1 (3.3%)	
**Mismatch repair**			0.4588			0.1328
Intact (MSS)	354 (80.6%)	85 (19.4%)		78 (83.9%)	15 (16.1%)	
Lost (MSI)	103 (77.4%)	30 (22.6%)		9 (64.3%)	5 (35.7%)	

*Fisher’s exact text (two-tailed).

Significant *p*-values values are in bold.

High vs. low NNMT expression levels showed no significant associations with the following parameters: patient gender, mucinous vs. non-mucinous histology, tumor differentiation (G1/G2 vs. G3), tumor location (left vs. right-sided), lymphovascular invasion status, perineural invasion status, or mismatch repair status (MSS vs. MSI). In early-stage cancers, high stromal NNMT expression was independent of patient age group at diagnosis (*p* = 0.4599), occurring in 19.0% of younger patients (≤70 years) vs. 21.7% of older patients (>70 years). In contrast, late-stage cancers displayed NNMT overexpression significantly more frequently (*p* = 0.0055) in older patients (62.5%) than in younger patients (21.7%).

NNMT expression showed significant correlation with cancer stage in both early and late-stage cohorts. In early-stage cancer cases, NNMT overexpression was found in 25/211 (11.8%) stage I and 90/361 (24.9%) stage II patients (*p* = 0.0001). In late-stage cancer cases, NNMT overexpression was found in 19/77 (24.7%) stage III and 1/30 (3.3%) stage IV patients. Comparison of different cancer stages suggests that the fraction of patients with stromal NNMT overexpression increases as early-stage tumors advance from stage I to stage II. The observed drop in NNMT-high stage IV patients could possibly be due to patients with NNMT-high cancers having an increased likelihood of dying before reaching stage IV, but this hypothesis would have to be tested in future work.

### NNMT expression versus patient survival

**Figure 3:**
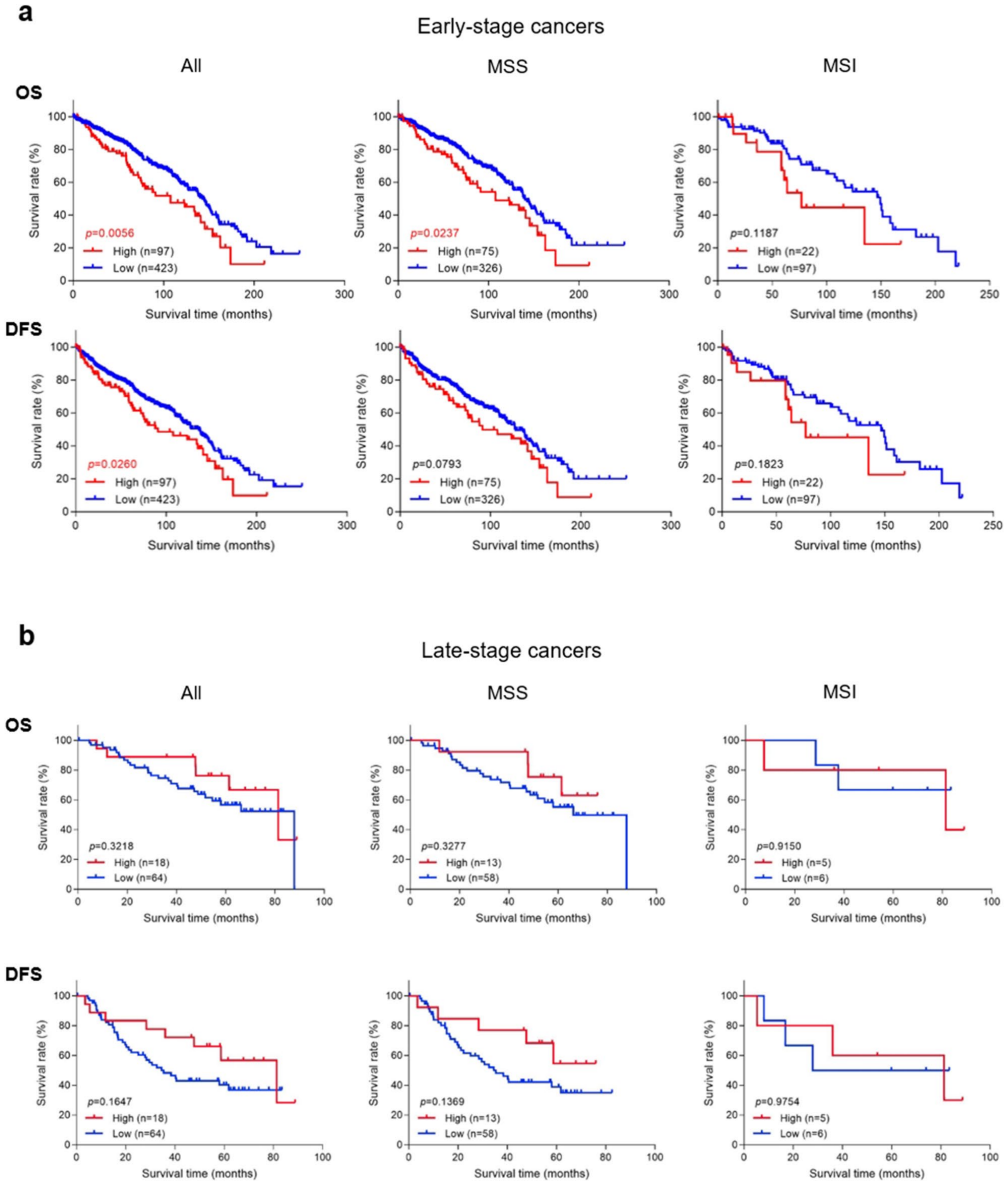
Overall survival (OS) or disease-free survival (DFS) of (**a**) early-stage and (**b**) late-stage colorectal cancer patients stratified by NNMT protein expression. High expression refers to cancer tissue with >50% stromal cells staining positively for NNMT protein. MSS, microsatellite stability; MSI, microsatellite instability.

To test the hypothesis whether high tumor stromal NNMT expression is associated with shorter patient survival, we performed Kaplan–Meier analyses of 520 early-stage and 82 late-stage patients whose survival data were available (Fig. 3). Both overall survival time and disease-free survival time were analyzed. The stage I and II patients of this study had been followed for a range of 0.2 to 392.5 months, with a mean follow-up time of 80.6 months and a median follow-up time of 72.5 months. The stage III and IV patients of this study had been followed for a range of 0.4 to 140 months, with a mean follow-up time of 51.2 months and a median follow-up time of 53.3 months. Among the 520 patients with early-stage colorectal cancer (Fig. 3a), NNMT overexpression was significantly associated with both shorter overall and disease-free survival (*p* = 0.0056 and *p* = 0.0260, respectively). Moreover, high NNMT levels were significantly associated with shorter overall survival in patients with the MSS subtype of colorectal cancer (*p* = 0.0237), with a similar trend observed for disease-free survival (albeit not statistically significant). Early-stage patients with the MSI subtype cancer also showed a trend for shorter survival when their NNMT protein levels were high, although the difference was not statistically significant. In contrast, NNMT protein expression levels in late-stage colorectal cancer patients did not show significant correlation with either overall or disease-free survival, irrespective of MSS or MSI subtype (Fig. 3b).

Based on these observations, we further analyzed the early-stage cohort and asked whether tumor stromal NNMT expression level or various clinicopathological parameters correlated with patient survival (Table 3). We performed both univariate and multivariate analyses. Higher patient age (>70 years), presence of lymphovascular invasion, presence of perineural invasion, higher tumor stage (II vs. I), and high stromal NNMT expression each significantly correlated with elevated death hazard ratios as measured by both overall and disease-free survival. Multivariate models of these parameters showed a similar trend.

**Table 3:** Univariate and multivariate analyses of patient survival in early-stage colorectal cancer.

Variables	Overall survival				Disease-free survival			
	Univariate		Multivariate		Univariate		Multivariate	
	HR* (95% CI)	*p*-value	HR* (95% CI)	*p*-value	HR* (95% CI)	*p*-value	HR* (95% CI)	*p*-value
Gender (male vs. female)	1.15 (0.87–1.52)	0.3333			1.28 (0.98–1.67)	0.0712		
Age (years) (> 70 vs. ≤ 70)	2.76 (2.08–3.71)	** < 0.0001**	2.69 (2.01–3.62)	** < 0.0001**	2.34 (1.79–3.07)	** < 0.0001**	2.26 (1.73–2.98)	** < 0.0001**
Tumor location (right vs. left)	1.30 (0.98–1.71)	0.0654			1.17 (0.90–1.52)	0.2455		
Histology (mucinous vs. other)	0.75 (0.42–1.25)	0.2816			0.80 (0.46–1.29)	0.3861		
Tumor differentiation (G3 vs. G1/2)	1.24 (0.70–2.03)	0.4366			1.12 (0.64–1.80)	0.6800		
Lymphovascular invasion	1.78 (1.19–2.58)	**0.0060**	1.78 (1.18–2.61)	**0.0073**	2.03 (1.41–2.85)	**0.0003**	1.97 (1.35–2.80)	**0.0006**
Perineural invasion	2.15 (1.19–3.59)	**0.0135**	1.57 (0.86–2.67)	0.1346	2.07 (1.17–3.38)	**0.0148**	1.45 (0.81–2.41)	0.2033
AJCC stage (II vs. I)	1.79 (1.34–2.44)	**0.0001**	1.44 (1.06–1.97)	**0.0206**	1.84 (1.39–2.47)	** < 0.0001**	1.54 (1.15–2.09)	**0.0037**
Mismatch repair (MSI vs. MSS)	1.09 (0.78–1.49)	0.6074			0.97 (0.70–1.32)	0.8628		
NNMT expression (high vs. low)	1.59 (1.13–2.19)	**0.0086**	1.38 (0.98–1.92)	0.0685	1.44 (1.03–1.96)	**0.0325**	1.23 (0.88–1.70)	0.2162

*HR hazard ratio of death, *CI* confidence interval.

Significant *p*-values are in bold.

### NNMT expression versus BRAF, KRAS, and PIK3CA mutations

**Figure 4:**
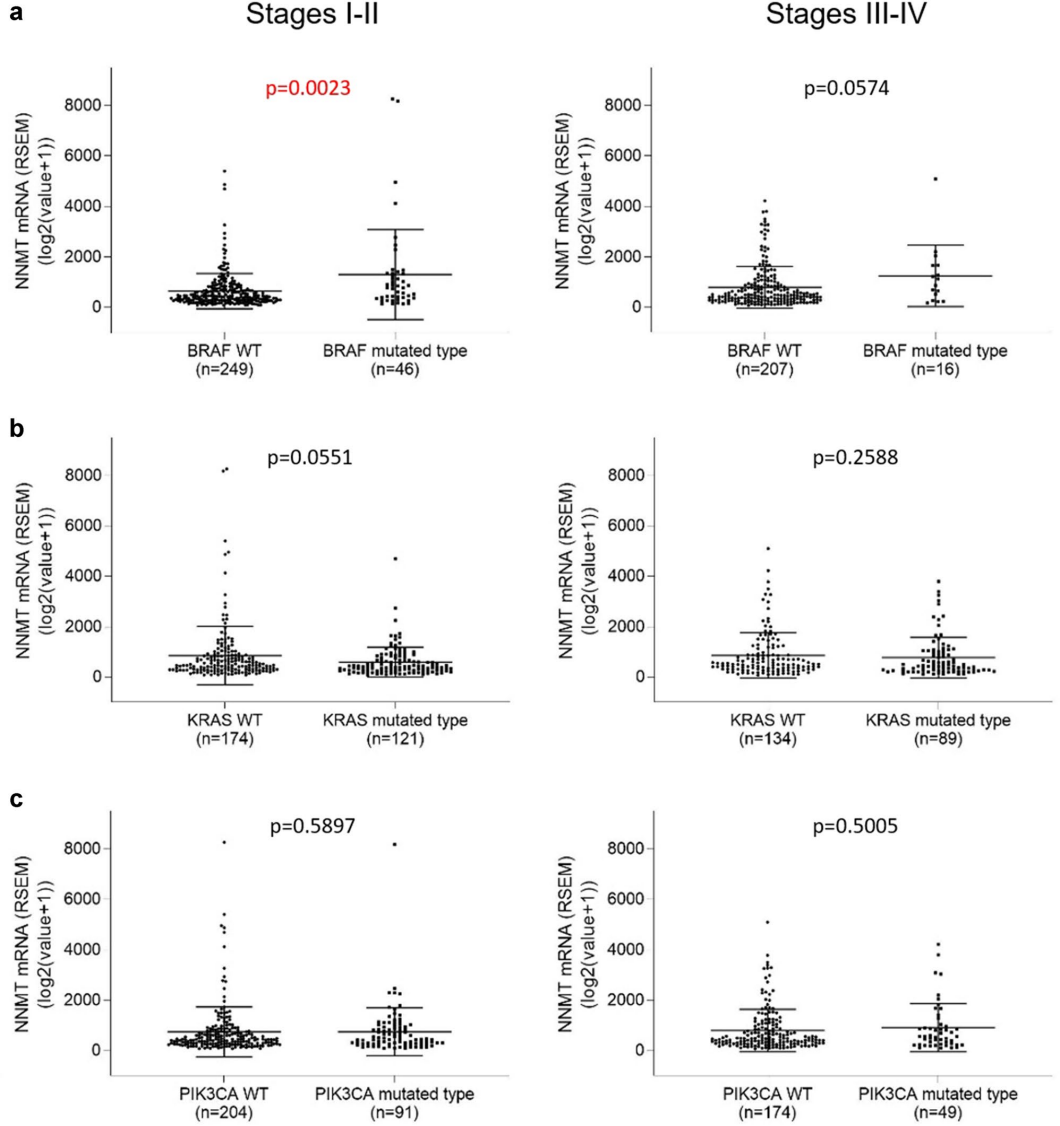
NNMT mRNA expression in colorectal adenocarcinomas as a function of wild type (WT) vs. mutated genomic status of (**a**) *BRAF*, (**b**) *KRAS*, or (**c**) *PIK3CA*. The left plots show early-stage cancers, while the right plots show late-stage cancers.

To understand whether there is a relationship between *NNMT* expression and known molecular markers of colorectal cancer, we analyzed gene transcript and protein expression profiles of TCGA and CPTAC colorectal cancer cohorts that comprise data from bulk tumor measurements of both tumor and tumor stroma^33–35^. Interestingly, in early-stage cancers, *NNMT* mRNA expression is significantly higher in *BRAF* mutated than in *BRAF* wild type cancers (Fig. 4a). In late-stage cancers, *NNMT* mRNA expression also appeared to be increased in *BRAF* mutated cancers relative to *BRAF* wild type, although the difference was not statistically significant. In contrast, neither *KRAS* nor *PIK3CA* mutated cancers showed differences in *NNMT* expression relative to respective wild type tumors (Fig. 4b,c).

In an attempt to understand NNMT interaction network in colorectal cancer, we examined related genes and proteins in the TCGA colorectal cancer cohort^33,34^. Based on protein domain homology, NNMT belongs to the class I-like SAM-binding methyltransferase NNMT/PNMT/INMT superfamily^38^, and INMT (indolethylamine N-methyltransferase) is an important paralog of NNMT. TCGA data reveals that *NNMT* mRNA expression is positively correlated with *INMT* but not with *PNMT*. Based on STRING interaction network analysis, NNMT interacts with ADK, APP, ATF6, BMF, BRD4, CDC37, or CRYZ; however, our analysis of TCGA colorectal cancer cohorts found no correlation between *NNMT* and these entities at mRNA expression level. Interestingly, we found that *NNMT* mRNA expression is negatively correlated with *NNMT* gene methylation (Supplemental Fig. 1c), suggesting that NNMT protein overexpression may lead to SAM methyl donor depletion causing a positive feedback loop of hypomethylation of the *NNMT* gene promoter locus, increased *NNMT* gene transcription, and NNMT protein overexpression.

### Increased NNMT expression in CIMP-high and MSI subtypes

**Figure 5:**
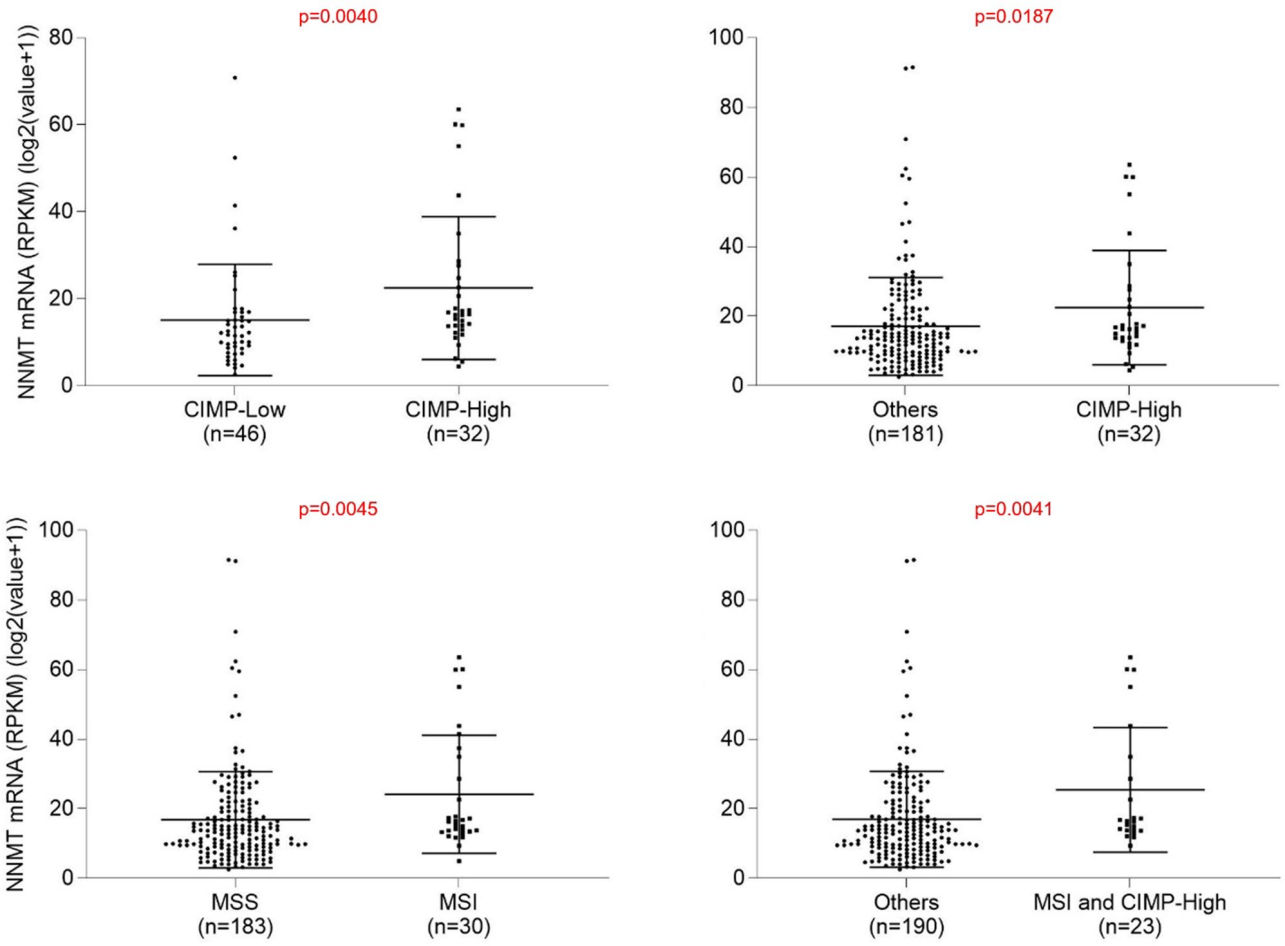
NNMT mRNA expression in colorectal adenocarcinomas as a function of CpG island methylator phenotype (CIMP) low or high and microsatellite stability (MSS) or microsatellite instability (MSI). Data for all stages I-IV are shown.

On the bases of CpG island methylation status, colorectal cancer is sub-classified into CIMP-high (cancers with extensive promoter methylation) and CIMP-low (cancers with less extensive promoter methylation). Since NNMT is a methyltransferase that uses the same methyl donor (SAM) as other methyltransferases, we asked whether its expression differed in CIMP-high and CIMP-low colorectal cancers. In the TCGA colorectal cancer cohort with available methylation data^33^, colorectal cancers were clustered into four subtypes, CIMP-high, CIMP-low, and two unknown clusters (“others”). Among these patients, *NNMT* mRNA expression is significantly higher in the CIMP-high phenotype than in the CIMP-low phenotype (*p* = 0.0040) (Fig. 5). Moreover, CIMP-high cancers have higher NNMT expression than other unclassified methylation-based clusters (*p* = 0.0187).

Colorectal cancers are typically classified into two subtypes: MSS (microsatellite stability) and MSI (microsatellite instability) phenotypes. We therefore asked whether *NNMT* expression differs between these two subtypes using the same TCGA cohort of colorectal cancer patients^33^. We found that *NNMT* expression was significantly higher in MSI subtype than in MSS subtype cancers (*p* = 0.0045) (Fig. 5). In addition, cancers that are both CIMP-high and of MSI subtype had significantly higher expression levels of *NNMT* than all other cancers (*p* = 0.0041).

## Discussion

We found that NNMT protein is weakly expressed in normal stromal cells of the lamina propria of benign colonic mucosa and can be significantly overexpressed in colorectal cancer tumor stromal cells but not in cancer cells (Figs. 1 and 2). With the goal of discovering markers for subclassification and risk stratification of early-stage colorectal cancer, our study investigated 572 patients with early-stage colorectal cancer and observed that NNMT protein overexpression is significantly correlated with both shorter overall and disease-free survival (Fig. 3). Subgroup analyses indicate that this survival difference is especially seen in the MSS subtype. Our findings are generally in line with a previous report of high stromal NNMT expression indicating a poor prognosis in a mixed cohort of early and late-stage colorectal cancers^5^. However, our examination of 107 cases of late-stage colorectal cancers did not support a correlation between NNMT expression and survival in patients with late-stage colorectal cancer (stages III and IV). Our data rather indicates that the outcome-predictive power of NNMT is strong in early-stage disease (stages I and II), which is also more clinically relevant because early-stage disease poses the therapeutic challenge of distinguishing low-risk from high-risk patients and avoiding overtreatment in the former and directing risk-reducing adjuvant therapy to the latter.

Based on the currently available TCGA and CPTAC datasets for which both gene and protein sequencing data are available, NNMT protein abundance is positively correlated with abundance of its mRNA transcript in colorectal cancer (Supplemental Fig. 1). A similar correlation was found in human liver, where individuals with high hepatic NNMT enzymatic activity had concordant high levels of both NNMT protein and *NNMT* mRNA levels and the converse was true for those with low NNMT activity^39^. Moreover, phenotypic differences of NNMT activity in tissue appear to be due to differences in steady-state mRNA levels rather than polymorphisms in the *NNMT* coding gene^39^. The positive correlation between mRNA and protein expression suggests that NNMT may be assessed as an outcome biomarker for early-stage colorectal cancer either at the protein or at the mRNA level, hence broadening its potential utility as a prognostic marker.

NNMT is a crucial enzyme in metabolism of nicotinamide and xenobiotic drugs, hence its overexpression may aid in risk stratification of drug treatment of colorectal cancer. For example, overexpression of NNMT in SW480 cells enhanced 5-fluorouracil resistance, whereas down regulation of NNMT in HT-29 cells diminished the drug resistance^40^. It will be interesting to explore in future studies whether NNMT overexpression can be used as a marker to guide adjuvant chemotherapy in colorectal cancer treatment.

Our study shows that higher levels of NNMT expression tend to be found in colorectal cancers with *BRAF* mutations or CIMP-high and/or MSI genomic background (Figs. 4 and 5). A previous study reported a CIMP-high colorectal cancer phenotype with underlying sporadic microsatellite instability and tight association with *BRAF* mutations but *KRAS* wild type^41^. Our current study adds NNMT as another marker to further define this subtype of colorectal cancer.

In summary, our study examined several cohorts of colorectal cancer patients and identified tumor stromal NNMT overexpression as a potential prognostic marker indicating poor clinical outcomes in early-stage colorectal cancer. Given its roles in drug metabolism, epigenetic regulation, and pan-cancer stromal expression, NNMT has the potential to serve as a screening marker for early detection and risk stratification for guiding therapeutic cancer management.

## Supplementary Information

Supplementary Information.

## Data Availability

All primary data from the manuscript is available from the corresponding author upon reasonable request.
