# Supplementary material for: Tumor stromal nicotinamide N-methyltransferase overexpression as a prognostic biomarker for poor clinical outcome in early-stage colorectal cancer
Source: Sci Rep. 2022 Feb 17;12:2767. doi: 10.1038/s41598-022-06772-w (PMC8854702; doi:10.1038/s41598-022-06772-w)
Supplement: Supplementary file 1 — Supplementary Information. [file 41598_2022_6772_MOESM1_ESM.pdf]

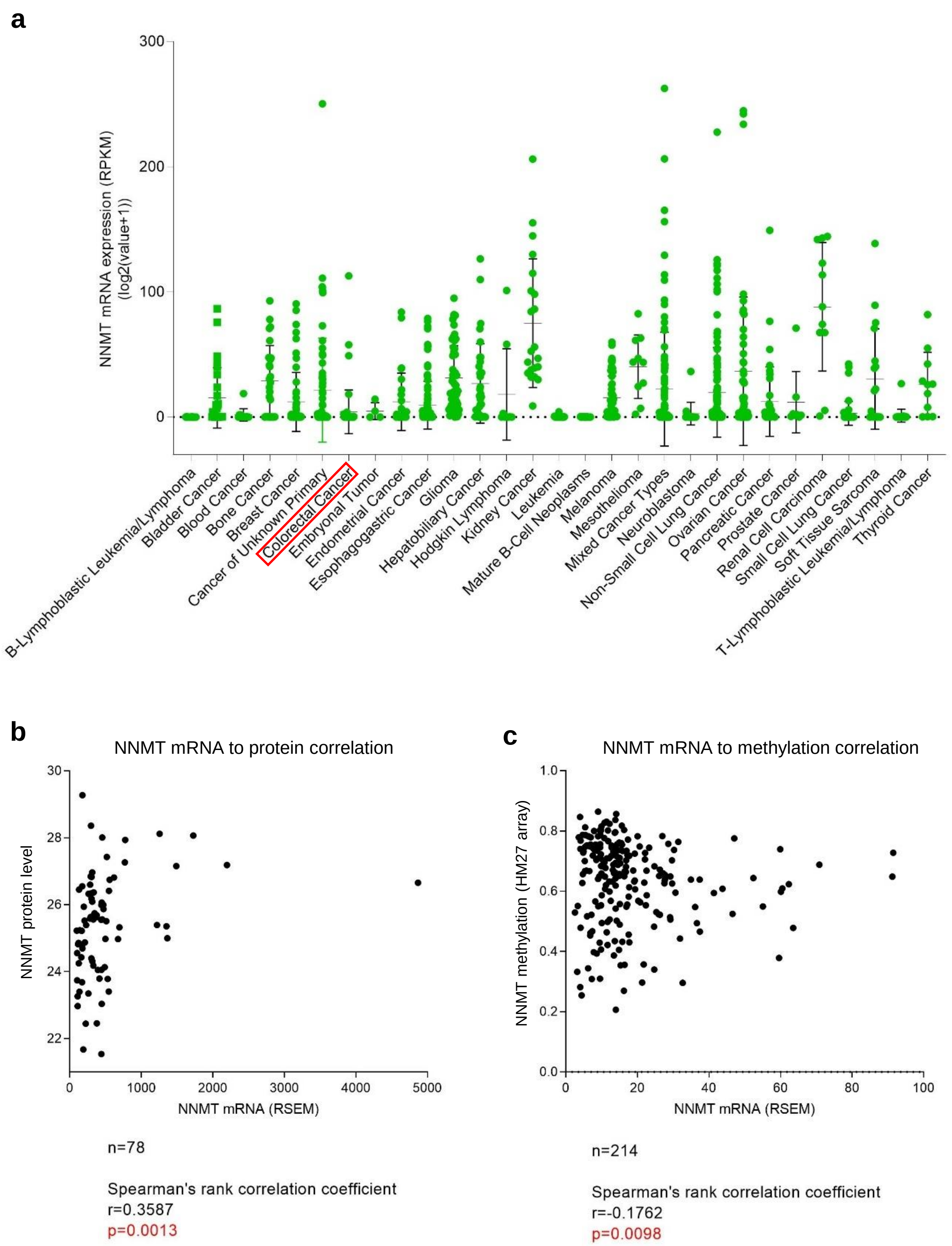

Supplementary Fig. 1. (**a**) NNMT mRNA expression levels across cell lines from multiple types of malignancies. (**b**) Correlation between NNMT mRNA expression and protein expression. (**c**) Weakly negative correlation between NNMR mRNA expression and NNMT gene methylation.
